# Comparative Studies on the Hepatoprotective Effect of White and Coloured Rice Bran Oil against Acetaminophen-Induced Oxidative Stress in Mice through Antioxidant- and Xenobiotic-Metabolizing Systems

**DOI:** 10.1155/2021/5510230

**Published:** 2021-04-27

**Authors:** Warunyoo Phannasorn, Arpamas Chariyakornkul, Phumon Sookwong, Rawiwan Wongpoomchai

**Affiliations:** ^1^Department of Biochemistry, Faculty of Medicine, Chiang Mai University, Chiang Mai 50200, Thailand; ^2^Functional Food Research Unit, Science and Technology Research Institute, Chiang Mai University, Chiang Mai 50200, Thailand; ^3^Rice and Cereal Chemistry Research Laboratory, Department of Chemistry, Faculty of Science, Chiang Mai University, Chiang Mai 50200, Thailand

## Abstract

Rice bran oil (RBO) comprises various nutrients and phytochemicals which exhibit several health benefits. There are no studies regarding the functional effects of different colours of RBO. This study was aimed to compare the constituents and antioxidant activities of white rice bran oil (WRBO) and coloured rice bran oil (CRBO). Each RBO showed similar free fatty acid profiles. However, greater amounts of vitamin E, phytosterols, carotenoids, and chlorophylls were found in CRBO, which had lower *γ*-oryzanol content than WRBO. Oxidative stress was induced in male mice by an overdose of acetaminophen (APAP) at 300 mg/kg body weight. The mice were then fed with RBO at the equivalent dose to 100 mg/kg body weight of *γ*-oryzanol three hours later and sacrificed six hours after APAP treatment. The administration of 100 mg *γ*-oryzanol equivalent in CRBO ameliorated APAP-induced hepatotoxicity in mice more strongly than 100 mg *γ*-oryzanol equivalent in WRBO, as evidenced by the significant reduction of serum ALT, hepatocellular necrosis, and hepatic lipid peroxidation. CRBO could improve xenobiotic-metabolizing and antioxidant enzyme activities, including glutathione *S*-transferase, superoxide dismutase, glutathione peroxidase, and glutathione reductase, and also increase mRNA expression of various antioxidant-responsive genes. Vitamin E, phytosterols, carotenoids, and chlorophyll might be the protective compounds in CRBO that alleviate APAP-induced hepatotoxicity through the interruption of APAP metabolism and the activation of antioxidant systems at both transcriptional and enzymatic levels. These findings might provide a protective role of CRBO on oxidative stress associated with several degenerative diseases.

## 1. Introduction

Oxidative stress results from an imbalance between the accumulation of free radicals and their elimination by antioxidant systems and causes damage to biomolecules such as lipids, DNA, and proteins [1]. Oxidative stress can accelerate the development of several degenerative diseases, such as diabetes mellitus, arthritis, cardiovascular diseases, neurodegenerative diseases, multiorgan failure, and cancer, and also plays a role in the aging process [2]. Several studies have shown that these diseases are associated with a decline of antioxidant potential resulting from an increase of free radical production along with a decrease of the concentrations of antioxidant enzymes and, also induction of an inflammatory state [3]. Antioxidants are a defence mechanism that protects biological systems from reactive oxygen and nitrogen species (RONS) produced by several processes [3]. Endogenous antioxidants include enzymatic and nonenzymatic pathways. Superoxide dismutase (SOD) converts the superoxide anion (O_2_^•-^) to hydrogen peroxide (H_2_O_2_) and oxygen. H_2_O_2_ is further catalyzed to water and molecular oxygen by catalase (CAT). Glutathione peroxidase (GPx) also catalyzes the breakdown of hydrogen peroxide and lipid hydroperoxides in the reaction with reduced glutathione (GSH) to form oxidized glutathione (GSSG). Glutathione reductase (GR) then reduces GSSG to regenerate GSH [4]. Additionally, nonenzymatic antioxidants including bilirubin, vitamin E, *β*-carotene, albumin, and uric acid are molecules that interact with free radicals and terminate the chain reactions [5]. Interestingly, exogenous antioxidants also neutralize RONS. They prevent lipid peroxidation of cell membranes such as vitamin C, vitamin E, phenolic compounds, acetylcysteine, and oil lecithin and so suppress pathological conditions [6]. Therefore, prevention strategies, particularly the consumption of dietary antioxidants in daily life, are recommended to reduce oxidative stress and its potential impact on disease.

Vegetable oil has been recommended for daily cooking due to its high monounsaturated and *ω*-6 polyunsaturated fatty acid contents and its positive health benefit on heart disease and cancer in clinical studies [7, 8]. Previous works have reported the health benefits of edible oils. Olive oil containing *β*-carotene, lutein, tocopherols, squalene, and phenolic compounds exhibited antioxidant, anti-inflammatory, antimicrobial, anti-atherogenic, and anti-carcinogenic characteristics [9]. Sesame oil and its components showed antioxidant and anti-inflammatory effects, which alleviate cardiovascular diseases [10]. Safflower oil, extracted from seeds by screw pressing, demonstrated potent antioxidant effects and antimicrobial activity against skin pathogens [11].

Rice bran oil (RBO) is extracted from the cuticle between the husk and the grain obtained from the rice milling process. The global market of RBO was US$ 4.04 billion in 2018 and will increase to US$ 5.12 billion by 2025. Although it is not widely used as cooking oil, its demand as a healthy oil in applications and functional foods has been steadily increasing [12]. RBO contains a great source of phytochemicals such as tocopherols and tocotrienols, *γ*-oryzanols, and phytosterols [13] providing health advantages including antioxidation, anti-inflammation, anticancer, and hypolipidemic properties [14, 15]. In Thailand, Jasmine rice is primarily grown and is the most widely exported white rice product while Riceberry, a newly registered organic purple rice variety obtained from crossbreeding, has become the most popular rice known for its health benefits. Several studies have reported antioxidant activities of white rice bran oil (WRBO), but few have studied coloured rice bran oil (CRBO), especially in *in vivo* experiments. Therefore, the present study was aimed to compare the contents of phytonutrients and phytochemicals between WRBO and CRBO and their antioxidant properties using acetaminophen (APAP)-induced oxidative stress and hepatotoxicity in a mouse model. This classical model has been used to investigate the hepatoprotective effect of therapeutic compounds [16].

## 2. Materials and Methods

### 2.1. Chemicals

APAP, *N*-acetylcysteine (NAC), thiobarbituric acid (TBA), 5,5′-dithiobis-2-nitrobenzoic acid (DTNB), xanthine, xanthine oxidase, nitroblue tetrazolium, GR, reduced GSH, phenylmethylsulfonyl fluoride (PMSF), *p*-nitrophenol (*p*-NP), *p*-nitrocatechol, and tert-butyl hydroperoxide (t-BHP) were purchased from Sigma Aldrich Corp. (St. Louis, MO, USA). Bovine serum albumin (BSA) and 1-chloro-2,4-dinitrobenzene (CDNB) were obtained from Thermo Fisher Scientific Inc. (Waltham, MA, USA). Trichloroacetic acid (TCA) and H_2_O_2_ were bought from Merck Millipore (Burlington, MA, USA). UDP-glucuronic acid was provided by the United States Biological (Salem, MA, USA). *β*-NADPH was obtained from Nacalai Tesque (Kyoto, Japan). Potassium chloride, magnesium chloride, sodium carbonate, and glycerol were purchased from VWR Corp. (Radnor, PA, USA). Ethylenediaminetetraacetic acid (EDTA) was obtained from Vivantis Technologies (Selangor Darul Ehsan, Malaysia). HPLC grade butanol, hexane, isopropanol, ethyl acetate, acetic acid, methanol, and acetonitrile were obtained from RCI Labscan Ltd. (Bangkok, Thailand).

### 2.2. RBO Preparation

White and coloured rice (*Oryza sativa* L.) obtained from Kurk Rice Mill (Chiang Rai, Thailand) were planted from October to December 2018 in Wiang Chai District, Chiang Rai, Thailand. White and coloured rice varieties used in this study were Khao Dawk Mali 105 and Riceberry, respectively. WRBO and CRBO were extracted using a mechanical screw press machine and then filtered by paper to remove precipitate. After that, RBO was further purified by press filtration. RBO was collected into a glass bottle with light protection and kept at room temperature until investigation.

### 2.3. Analysis of Phytonutrients and Phytochemicals

The free fatty acid profile was analysed by the Institute of Food Research and Product Development, Kasetsart University, Bangkok, Thailand, using a gas chromatography-mass spectrometry technique according to an in-house method based on the Compendium of Methods for Food Analysis Thailand, 1st edition, 2003.

The assessment of *γ*-oryzanols was performed according to the method of Pintha et al. [17]. The HPLC system consisted of an Agilent HPLC 1260 connected to a diode array detector (Model G1311 C; Agilent Technologies, Santa Clara, CA, USA). RBO dissolved in ethanol was analysed using reversed-phased HPLC with a C18 column (Inertsil ODS-3 250 × 4.6 mm, 5 *μ*m; GL Sciences Inc., Tokyo, Japan). The mobile phase included methanol and acetonitrile (65 : 35) with isocratic elution and a flow rate of 1.0 ml min^–1^. A wavelength of 325 nm was used to monitor *γ*-oryzanols.

The determination of total vitamin E or tocols was performed by HPLC using a modified method of Huang et al. [18]. The system consisted of an Agilent HPLC 1100 connected to a fluorescence detector (Model 1046A; Hewlett Packard, Palo Alto, CA, USA). Twenty microliters of sample were injected into a normal-phase silica column (VertiSepTM UPS 4.6 mm × 250 mm, 5 *μ*m; Vertical Chromatography Co., Ltd., Nonthaburi, Thailand). The mobile phase was an isocratic gradient of hexane: isopropanol: ethyl acetate: acetic acid (97.6 : 0.8 : 0.8 : 0.8, *v*/*v*/*v*/*v*) with a flow rate 1 ml min^–1^, at room temperature. Tocols were fluorescently detected with the excitation at 294 nm and emission at 326 nm.

Phytosterols were analysed following the method of Pokkanta et al. [13]. The HPLC system consisted of an Agilent HPLC 1100 connected to a diode array detector (Model G1315 A; Agilent Technologies, Santa Clara, CA, USA). Each sample (5 *μ*L) was injected into a Kinetex PFP column (4.6 × 250 mm, 5 *μ*m, Phenomenex, Inc., Torrance, CA, USA) and gradually eluted by a mixed methanol and water solution with a flow rate of 1.0 ml min^−1^ for 30 min. Each form of phytosterols was detected at the wavelength of 210 nm.

Carotenoids and chlorophyll contents were determined using spectrophotometry. RBO was dissolved in 10% hexane (*w*/*v*). Carotenoid content was determined using the absorbance at 446 nm, while chlorophyll content was measured using the absorbance at wavelengths of 630, 670, and 710 nm and computed using the equations described by Pohndorf et al. [19].

### 2.4. Animals and Experimental Protocol

**Figure 1:**
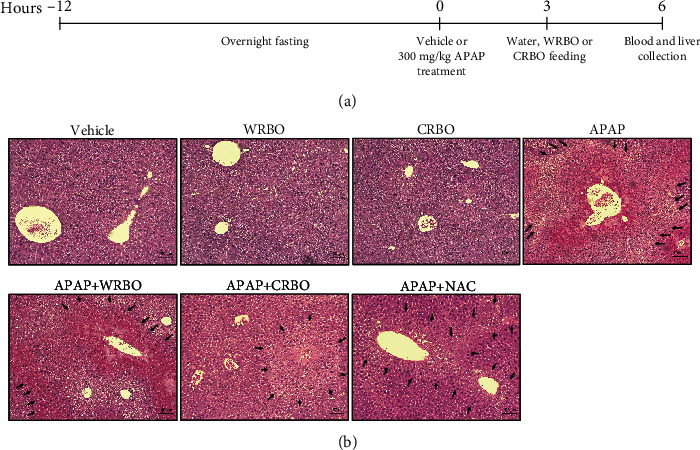
(a) The experimental protocol of RBO treatment in APAP-induced mice. Fasted male mice were orally administered with vehicle or 300 mg kg–1 BW of APAP. Three hours later, mice were administered with distilled water, WRBO, CRBO, or NAC and then euthanized six hours after APAP treatment. (b) Hepatic histomorphological changes in APAP-treated mice after RBO treatment by H&E staining under light microscopy. Micrographs are shown at 100x magnification. Arrows indicate necrotic areas. APAP: acetaminophen; WRBO: white rice bran oil 105; CRBO: coloured rice bran oil; NAC: *N*-acetylcysteine.

Ten-week-old male Mlac: ICR mice, weighing approximately 40–50 g, were purchased from the National Laboratory Animal Center, Mahidol University, Nakhon Pathom, Thailand. All mice were kept in cages following standard conditions including temperature at 25°C under a dark-light cycle and were fed ad libitum with basal diet and drinking water. An experimental protocol was approved by The Animal Ethics Committee of the Faculty of Medicine, Chiang Mai University (07/2561). Forty-two mice were randomly divided into seven groups, six mice per group. All mice were fasted overnight for 12–15 hours prior to APAP treatment. Groups 1–3 were orally administrated with 0.9% warmed saline (a vehicle), while groups 4–7 were orally fed with 300 mg kg^–1^ body weight (BW) of APAP. After APAP induction for three hours, groups 1 and 4 were then fed with 15 ml kg^–1^ BW of distilled water. Groups 2 and 5 were orally administrated with WRBO, while groups 3 and 6 were treated with CRBO at the equivalent dose to 100 mg kg^–1^ BW of *γ*-oryzanols. Group 7 was fed with 1200 mg kg^–1^ BW of NAC as a positive drug control (Figure 1(a)). After that, mice were euthanized six hours after APAP treatment under anaesthesia by intraperitoneal injection of 200 mg kg^–1^ BW of sodium pentothiol. Blood was collected from the hepatic portal vein and then centrifuged at 3500 g and 4°C to obtain serum for alanine aminotransferase (ALT) analysis using an automated analyser by the Small Animal Hospital, Faculty of Veterinary Medicine, Chiang Mai University. After liver tissues were excised and weighed, the cut portions were stored at –80°C until further biochemical evaluation or fixed in 10% phosphate-buffered formalin for histopathological analysis by haematoxylin and eosin (H&E) staining.

### 2.5. Preparation of Liver Homogenate, Cytosol, and Microsome

The liver tissue was homogenized in an ice-cold homogenizing buffer containing 1.15% *w*/*v* KCl and 0.25 mM PMSF. The homogenate was centrifuged at 10,000 rpm for 20 min at 4°C. The supernatant was collected and then centrifuged at 100,000 g at 4°C for 60 min to obtain a cytosolic supernatant and a microsomal pellet. The microsomal fraction was washed in homogenizing buffer and resuspended in microsomal suspension buffer pH 7.4, containing 30% (*v*/*v*) glycerol and 1 mM dithiothreitol. The cytosol and microsomal fractions were kept at –80°C until analysis. The protein content was measured by the Lowry method using BSA as a standard.

### 2.6. Liver Thiobarbituric Acid Reactive Substance Level

Lipid peroxidation in the liver was measured using a thiobarbituric acid reactive substance (TBARS) assay as previously described by Noeman et al. [20]. Liver homogenate was preincubated with TCA for 5 min and then centrifuged at 6,000 g, 4°C for 20 min. The supernatant was incubated with TBA in boiling water for 10 min. The resulting pink chromogen was extracted in butanol. The mixture was centrifuged at 3000 g for 10 min, and the supernatant was monitored using the optimal density at a wavelength of 532 nm.

### 2.7. Glutathione Content

Total GSH was determined using a GSH recycling system [21]. Liver homogenate was centrifuged at 14,000 rpm, 4°C for 20 min. The resulting upper part was mixed with the reaction mixture consisting of 6 unit ml^–1^ of GR and 1.5 mg ml^−1^ of DTNB at 25°C for 5 min and then 40 mg ml^−1^ of *β*-NADPH was added. The yellow colour was developed, and the absorbance was measured kinetically at 405 nm for 5 min. Total GSH was calculated using a calibration curve and expressed as nmol mg^–1^ protein. For GSSG measurement, the liver cytosol was treated with 4-vinylpyridine for 1 h before adding the reaction mixture. The GSSG content was calculated from the standard curve and expressed as nmol mg^–1^ protein. Reduced GSH content was obtained by subtracting the levels of GSSG from total GSH.

### 2.8. Activities of Xenobiotic-Metabolizing Enzymes

Cytochrome P450 2E1 (CYP2E1) activity was determined by measurement of *p*-nitrocatechol formation from *p*-NP. The liver microsome was added to the reaction mixture containing assay buffer, *p*-NP, NADP^+^, D-glucose-6 phosphate, MgCl_2_•6H_2_O, and glucose-6 phosphate dehydrogenase and then incubated for 30 min at 37°C. The reaction was halted with 10% TCA and was centrifuged at 10,000 g for 5 min. The supernatant was neutralized with NaOH. The end product, 4-nitrocatechol, was monitored at a wavelength of 535 nm [22].

UDP-glucuronosyl transferase (UGT) activity was determined using a modified method of Chariyakornkul et al. [23]. The reaction mixture containing *p*-NP, Tris–HCl buffer at pH 8.5, and MgCl_2_ was preincubated with microsomal fraction at 37°C for 5 min. UDP-glucuronic acid was added to the reaction mixture, and then, this was incubated for 20 min at 37°C. After stopping the reaction with 10% TCA, the mixture was then centrifuged at 10,000 g for 5 min. The supernatant was alkalinized with 0.5 M NaOH. The conjugated *p*-NP was quantified at 405 nm. UGT activity was expressed as pmol *p*-NP conjugate formed min^–1^ mg^–1^ protein.

Glutathione *S*-transferase (GST) activity was analysed using the method of Chariyakornkul et al. [23]. The reaction mixture contained potassium phosphate buffer at pH 6.5, GSH, CDNB, and the cytosolic fraction. The reaction was performed at 37°C for 90 s. Then, the absorbance at 340 nm was recorded and calculated using an extinction coefficient of 9.6 M^–1^ cm^–1^.

### 2.9. Antioxidant Enzyme Activities

To determine SOD activity [24], the cytosol was preincubated with a reaction mixture containing xanthine, nitroblue tetrazolium, EDTA, sodium carbonate, and BSA at 25°C for 30 min. The reaction was stopped using CuCl_2_ after adding xanthine oxidase and further incubated at 25°C for 20 min. The production of formazan was determined at a wavelength of 560 nm. The SOD activity was expressed as unit mg^–1^ protein.

To determine CAT activity [25], the reaction mixture consisting of H_2_O_2_ and phosphate buffer (pH 7.0) was mixed with the cytosolic sample. CAT activity was determined by the decrease in the absorbance of H_2_O_2_ at a wavelength of 240 nm and expressed as nmol of H_2_O_2_ min^–1^ mg^–1^ protein.

To determine GPx activity [26], the reaction mixture containing Tris–EDTA buffer (pH 8), GSH, *β*-NADPH, t-BHP, and GR was mixed with the cytosolic fraction. The decrease of *β*-NADPH was proportional to GPx activity at an absorbance 340 nm. The GPx activity was expressed as unit mg^–1^ protein.

To determine GR activity [27], the reaction mixture contained liver cytosol, GSSG, *β*-NADPH, and potassium phosphate buffer at pH 7.0. The decrease in absorbance of *β*-NADPH at 340 nm was determined spectrophotometrically at 37°C. Specific activity was expressed as unit mg^–1^ of protein.

### 2.10. Determination of Hepatic Antioxidant and Xenobiotic-Metabolizing Enzymes and Transcription Factor Involving APAP Metabolism in mRNA Levels

Frozen liver tissue was thawed, and mRNA was extracted using Purezol reagent (Bio-rad, Hercules, CA, USA). Extracted mRNA was reverse transcribed to cDNA using a high-capacity cDNA reverse transcription kit (Applied Biosystems, Foster City, CA, USA). The RNA levels of antioxidant and xenobiotic-metabolizing genes including SOD1, CAT, GCLC, GSTA, GPX1, and also GSR and nuclear factor erythroid-derived 2-like2 (NFE2L2) were evaluated by quantitative reverse transcription polymerase chain reaction (qRT-PCR) using specific primers with a SensiFAST™ SYBR® Lo-ROX Kit reagent (Bioline, London, UK) in the QuantStudio™ 6 Flex System (Thermo Fisher Scientific, Waltham, MA, USA).  Table 1 demonstrates the primer sequences for each target gene. The amplification procedure was run at 95°C for 2 min, followed by 40 cycles at 95°C for 5 s and 60°C for 35 s. RNA expression levels of target genes were normalized with *β*-actin mRNA and then presented as fold induction using the ∆∆^Ct^ method [28].

### 2.11. Statistical Analysis

All data are represented as mean ± SEM. Statistical analysis was performed using Statistical Package for the Social Sciences (SPSS) version 17.0 software (SPSS Inc., Chicago, IL, USA). The significant difference between groups in each experiment was analysed using one-way analysis of variance (ANOVA) followed by least significant difference (LSD) tests. Values of *p* < 0.05 were considered to be statistically significant.

## 3. Results

### 3.1. Phytonutrient and Phytochemical Composition in RBO

**Figure 2:**
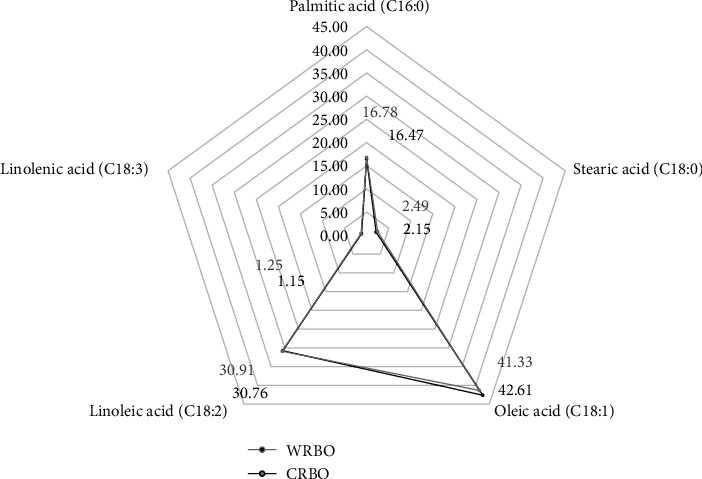
Composition of main free fatty acids in RBO analysed by gas chromatography-mass spectrometry (percentage of oil content).

First, we compared the main components in WRBO and CRBO. As summarized in Figure 2, free fatty acid profiles of WRBO and CRBO were very similar in the amounts of saturated fatty acids, monounsaturated fatty acids, and polyunsaturated fatty acids. The main free fatty acids were oleic acid, followed by linoleic acid (*ω*-3) and palmitic acid, respectively. Phytochemicals commonly found in RBO are shown in Table 2. With the exception of *γ*-oryzanols, the amounts of total vitamin E, phytosterols, carotenoids, and chlorophylls in CRBO were greater than in WRBO. The total *γ*-oryzanol content in WRBO (66.50 mg g^–1^ RBO) was higher than in CRBO (56.74 mg g^–1^ RBO), with *β*-sitosteryl ferulate being a major *γ*-oryzanol. While CRBO contained higher total amounts of vitamin E (1,467.24 *μ*g g^–1^ RBO) than WRBO (1,151.41 *μ*g g^–1^ RBO), *γ*-tocotrienol is a prominent vitamin E isoform in both RBOs. All isoforms of vitamin E were higher in CRBO than WRBO, with the exception of *α*-tocopherol and *α*-tocotrienol. Moreover, phytosterols, carotenoids, and chlorophylls, mainly found in edible oils, were found in large amounts in CRBO.

### 3.2. Hepatoprotective Effect of RBO on APAP-Induced Hepatotoxicity in Mice

As APAP overdose causes liver damage, the changes in parameters of liver function after APAP induction were examined. APAP overdose significantly increased the levels of serum ALT in the APAP-treated group by 677-fold when compared to the control (Table 3). WRBO treatment slightly decreased serum ALT level (*p* > 0.05). While treatment with CRBO and NAC in mice three hours after APAP administration significantly decreased ALT level when compared to the APAP-treated alone group. The administration of RBO alone had no significant effect. The effect of various RBOs on the oxidative stress markers consisting of malondialdehyde (MDA), total GSH content, and GSSG/GSH ratio are also shown in Table 3. An increased MDA level and decreased total GSH content in the livers of APAP-fed mice were observed. RBO treatment alone did not affect MDA levels. However, the administration of APAP, followed by CRBO or NAC feeding, could attenuate the increase of MDA levels observed in the APAP group (*p* < 0.05). The administration of CRBO and NAC restored liver total GSH levels in the APAP-treated group, which NAC is the most effective in GSH regeneration. Unexpectedly, the ratio of GSSG/GSH was not significantly different in all groups. These results show the hepatoprotective properties of CRBO in APAP-induced liver damage.

### 3.3. Effect of RBO on Mice Hepatic Histomorphological Changes in APAP-Induced Mice

Representative images of the H&E-stained liver sections are shown in Figure 1(b). The livers of the control group showed the normal appearance of hepatic structure with normal central vein and hepatic lobule. The central vein was surrounded by normal hepatic cords and sinusoids. Similarly, the sections of liver tissue from mice treated with RBO alone had similar hepatic histomorphological patterns. In contrast, the livers of the APAP-treated alone group presented a large area of hepatocellular necrosis and inflammation. The congested central vein was filled with red blood cells and enclosed by hepatic cords with sinusoidal dilatation. Pyknosis and neutrophilic and lymphocytic infiltration were also markedly detected. Interestingly, administration of CRBO or NAC after APAP treatment showed an amelioration of APAP hepatotoxicity with the presence of a mild necrotic area with the recovery of liver architecture.

### 3.4. APAP-Metabolizing and Antioxidant Enzyme Activities in the Liver

**Figure 3:**
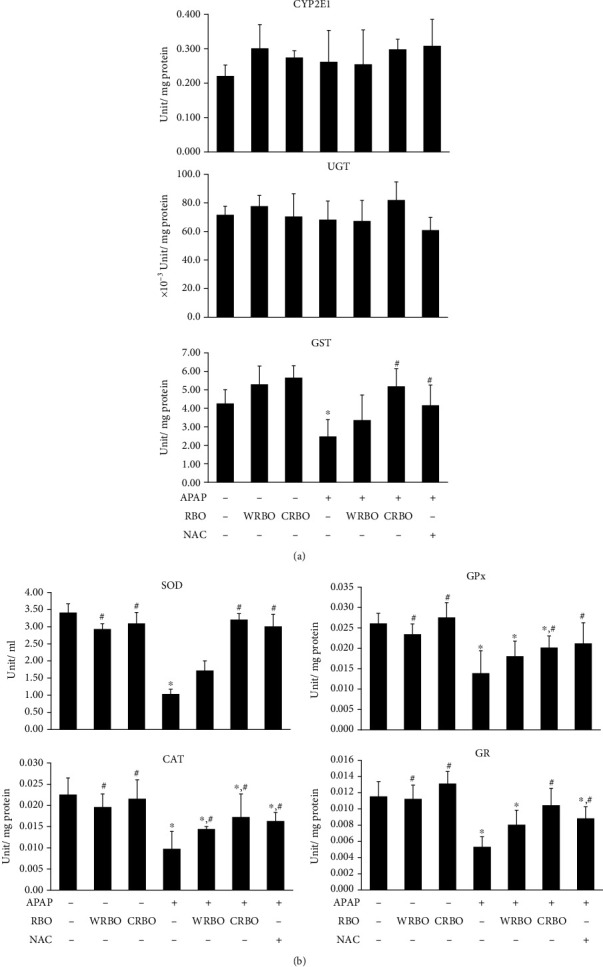
Effect of RBO administration on the activities of hepatic xenobiotic-metabolizing and antioxidant enzymes in mice. (a) APAP-metabolizing enzyme activities including CYP2E1, UGT, and GST. (b) Antioxidant enzyme activities including SOD, CAT, GPx, and GR. Results are expressed as mean ± SEM (*n* = 6). ^∗^*p* < 0.05 significantly different from vehicle. ^#^*p* < 0.05 indicates significant difference from APAP. CYP2E1: cytochrome P450 2E1; UGT: UDP-glucuronosyltransferase; GST: glutathione *S*-transferase; SOD: superoxide dismutase; CAT: catalase; GPx: glutathione peroxidase; GR: glutathione reductase; APAP: acetaminophen; RBO: rice bran oil; WRBO: white rice bran oil; CRBO: coloured rice bran oil; NAC: *N*-acetylcysteine.

The beneficial effects of RBO on detoxifying and antioxidant systems are shown in Figure 3. The changes of APAP-metabolizing enzyme activity including CYP2E1 and UGT could not be observed in all treatments. However, the activity of GST was significantly (*p* < 0.05) decreased in APAP-treated mice compared to the control. After CRBO or NAC administration, the activity of this enzyme was increased significantly (*p* < 0.05) three hours later compared to the APAP-treated alone group (Figure 3(a)). As can be seen in Figure 3(b), the data demonstrated that treatment with APAP caused a significant decrease in the activities of hepatic antioxidant enzymes including SOD, CAT, GPx, and GR compared with those of the control mice. Administration of RBO alone caused insignificant increase of the activity of each enzyme in the liver compared to the control mice. In addition, the administration of CRBO or NAC significantly increased SOD, CAT, GPx, and GR activity in the liver compared with the APAP-treated alone group.

### 3.5. Expression of APAP-Metabolizing and Antioxidant Response Genes in the Liver

**Figure 4:**
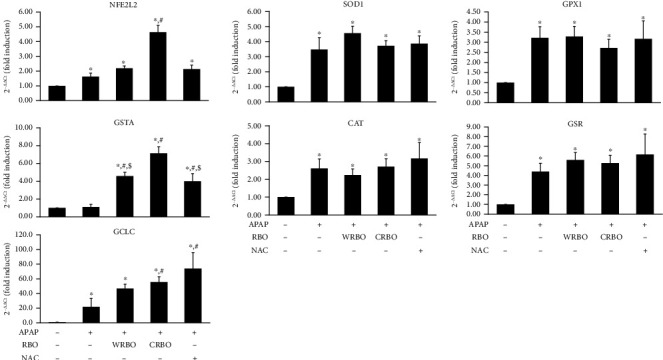
Effect of RBO administration on mRNA levels of antioxidant responsive genes in the livers of APAP-treated mice. Results are expressed as mean ± SD (*n* = 6). ^∗^*p* < 0.05 significantly different from vehicle. ^#^*p* < 0.05 indicates significantly different from APAP. ^$^*p* < 0.05 significantly different from APAP+CRBO. NFE2L2: nuclear factor erythroid-derived 2-like2; GSTA: glutathione *S*-transferase A1; GCLC: glutamate-cysteine ligase catalytic subunit; SOD1: superoxide dismutase 1; CAT: catalase; GPX1: glutathione peroxidase 1; GSR, glutathione reductase; APAP: acetaminophen; RBO: rice bran oil; WRBO: white rice bran oil; CRBO: coloured rice bran oil; NAC: *N*-acetylcysteine.

Treatment with CRBO or NAC improved GST, SOD, CAT, GPx, and GR activities in the livers of APAP-treated mice. Thus, qRT-PCR analysis for gene expression of these antioxidant enzymes and transcription factors was performed and the results are shown in Figure 4. The expression of transcription factor NFE2L2 and antioxidant responsive genes including GSTA1, GCLC, SOD1, CAT, GPX1, and GSR were increased in mice fed with APAP (*p* < 0.05). The administration of WRBO, CRBO, or NAC in APAP-treated mice did not affect the expression of SOD1, CAT, GPX1, and GSR genes when compared to APAP-administered alone group. Treatment with WRBO, CRBO, and NAC after APAP induction showed the increased mRNA levels of GSTA when compared to APAP-treated alone group. GSTA mRNA was highly expressed in the APAP with CRBO-fed group. Furthermore, the mRNA level GCLC (catalytic unit of glutathione biosynthesis enzyme) was significantly increased in the mice treated with APAP together with CRBO or NAC compared to the group treated with APAP alone. Interestingly, CRBO treatment enhanced the NFE2L2 gene for the oxidative response-transcription factor (*p* < 0.05) at the mRNA level in APAP-induced mice. These results show that CRBO improved the antioxidant properties not only at the level of enzyme activities, but also in transcriptional level.

## 4. Discussion

Under abnormal environmental stress, the overproduction of RONS causes significant damage to important biomolecules. This damage may occur to cell structures and functions and has a potential impact on several pathological effects. Natural and synthetic antioxidants could reduce the incidence of oxidative stress-mediated diseases. The study of the antioxidant activities from various bioactive compounds such as dietary fiber, phenolic compounds, tocols, phytic acid, and phytosterols is of growing interest in clinical trials [29]. Comparative studies showed that coloured rice bran contained higher amounts of phytochemicals such as *γ*-oryzanols and vitamin E than white rice bran and also showed higher antioxidant activities [30, 31]. Coloured rice bran extract showed greater antiproliferative activity than brown rice bran extract against MCF-7 and MDA-MB-231 breast cancer cell lines [32]. In addition, defatted sticky purple rice bran extract could inhibit preneoplastic lesion formation of carcinogen-induced hepatocarcinogenesis in rats with five weeks of treatment compared with white rice bran extract by attenuation of inflammation and cell proliferation [33]. Even though rice bran has been reported, studies comparing RBOs are few. Sengupta et al. [34] reported an antioxidative effect of RBO in arsenite-induced oxidative stress in rats by improvement of CAT, SOD, GPx, and GR activity and the inhibition of lipid peroxidation. The present study shows that RBO obtained from coloured rice (Riceberry) also reduced hepatotoxicity and exhibited antioxidant activities in mice given an overdose of APAP.

APAP or *N*-acetyl-*p*-aminophenol is commonly used to treat pain and fever [35]. APAP usage at recommended therapeutic concentrations is important because an overdose of APAP can cause oxidative stress and then severe liver damage, resulting in acute liver failure. APAP overdose-induced liver hepatotoxicity is a well-known drug-induced liver injury. It is the most common cause of acute liver failure in several countries, replacing viral hepatitis [36]. Extensive oxidative stress is a characteristic of APAP hepatotoxicity during APAP metabolism. Detoxification of APAP is mainly catalyzed in the liver by UGT and sulfotransferases (SULT) at therapeutic doses. However, APAP overdose is metabolized by CYP2E1 to the active metabolite *N*-acetyl-*p*-benzoquinone imine (NAPQI) and excreted as the glutathione conjugated form by GST. Excess NAPQI can deplete hepatic GSH stores and adduct target proteins through binding their cysteine groups, especially mitochondrial proteins. Impairment of the mitochondrial electron transport system causes ROS formation, mitochondrial permeability transition (MPT), oxidative stress, and finally oncotic necrosis [16].

The classical mechanism of liver tissue damage by hepatotoxins such as APAP is hepatocellular enzyme leakage as diagnosed by increased serum ALT [37]. An abnormal ALT was observed in APAP-treated mice in this study. CRBO reduced the increased ALT activities from an overdose of APAP. The hepatoprotective effect of CRBO was similar to NAC treatment, which is widely used as a therapeutic drug for liver failure from APAP poisoning [38]. Furthermore, liver necrosis and failure from APAP overdose could be observed by histopathological alteration. Consistent with the ALT level result, administration of 300 mg kg^–1^ BW of APAP caused markedly developed hepatocellular necrosis in cross sections of liver tissue, similar to those observed previously in mice [39]. Administration of CRBO gave liver sections with nearly normal architectures and normal hepatocytes in APAP-induced mice. Lipid peroxidation is one consequence of oxidative stress promoted by high intracellular concentrations of ROS. MDA is a well-known biomarker of oxidative stress produced by the reaction of polyunsaturated fatty acid peroxidation [40]. Overproduction of MDA is correlated with APAP-induced tissue damage. The results showed that a significantly increased MDA level in the livers of APAP-treated mice was decreased by administration of CRBO or NAC, indicating their properties to inhibit lipid peroxidation. These findings suggested the possibility of using the hepatoprotective effect of CRBO as shown by the significant recovery from hepatic necrosis, concurrently with decreased serum ALT levels.

At therapeutic doses, APAP is mostly converted to inactive glucuronide and sulfated conjugates by UGT and SULT, respectively. APAP also is marginally metabolized by cytochrome P450 to form the highly reactive species NAPQI, which is readily detoxified with GSH conjugation by glutathione *S*-transferase under normal conditions. However, saturation of detoxification pathways by APAP overdose causes the depletion of GSH and overproduction of NAPQI, mostly metabolized by CYP2E1 which directly binds to cellular biomolecules. The binding on cysteine residues of protein in mitochondria can cause mitochondria dysfunction including electron transport inhibition, mitochondrial oxidative stress, and onset mitochondrial permeability transition. This results in decreased energy production and finally cellular necrosis [41]. The results show that the activity of UGT and CYP2E1 did not alter in both APAP-treated alone and APAP treated with RBO groups when compared to the control group. Glucuronidation causes saturation at highly toxic doses, while CYP2E1 activity was raised 1 hour after APAP administration [41, 42]. Our data indicate UGT and CYP2E1 might not be involved in the hepatoprotective effect of RBO. However, GST could increase the activity in APAP-treated mice after the administration of CRBO or NAC 3 hours later. GST expressed higher activity than the other xenobiotic-metabolizing and antioxidant enzymes due to the lower *K*_*m*_ for NAPQI (15 *μ*M), which suggested a critical role of GST in NAPQI detoxification by GSH to form APAP–GSH adducts [43]. This was also related to total GSH contents. GSH is a crucial cellular antioxidant which is maintained at a certain level in cells [44]. The quantity of total GSH in mice livers was decreased after 1 hour of overdose of APAP at 300 mg/kg bw until 6 hours and was then steadily regenerated [39]. Our results confirmed that hepatic GSH levels had significantly decreased after 6 hours of APAP administration when compared to the vehicle group. Treatment with CRBO or NAC increased GSH levels 3 hours after APAP treatment. As expected, NAC, a precursor for GSH biosynthesis, might allow more GSH for detoxification of NAPQI. In addition, the hepatic GSSG/GSH ratio in mice increased significantly after 6 hours, which might be attributed to oxidative stress in mouse livers [39]. Unfortunately, the ratio of GSSG : GSH in this study did not show any substantial changes in each group. Glutathione might be destroyed by acetaminophen rather than oxidized by free radicals. Interestingly, the results suggest that CRBO treatment could accelerate GSH recovery and then promote GST activity in APAP-treated mice to play a critical role in NAPQI detoxification by GSH to form APAP–GSH adducts. Therefore, one mechanism of hepatoprotection from CRBO could involve APAP metabolism by conjugation with GSH, leading to reduction of oxidative stress.

SOD, CAT, GPx, and GR are the main endogenous enzymatic defence systems for preventing damage by oxidative stress of aerobic cells [4]. The results show that each antioxidant enzyme had decreased activity in the APAP-treated mice group due to excessive production of ROS. This suggested they had a critical role in the prevention of APAP hepatotoxicity. Treatment with CRBO and NAC could recover levels of SOD, CAT, GPx, and GR activities in APAP-induced mice when compared to the APAP-treated alone group. SOD had the highest activity compared with the activities of the other antioxidant enzymes. The reaction of superoxide with SOD is first order and has the greatest *k*_cat_/*K*_*m*_ with respect to superoxide concentration for the defence of living cells exposed to oxygen [45]. The results show that CRBO has antioxidant properties leading to lowering the oxidative stress induced by APAP.

Nuclear factor erythroid 2-related factor 2 (Nrf2) is a key transcription factor encoded by the NFE2L2 gene that controls the expression of antioxidants and phase II of xenobiotic-metabolizing genes to regulate both normal and oxidative stress conditions. ROS activate the dissociation of Nrf2 from Keap1 and consequently translocate and bind to the antioxidant response element (ARE) in the nucleus, inducing the expression of ARE-responsive genes such as GSR, GCLM, GCLC, GPX, HMOX, G6PD1 [46]. CRBO administration significantly increased mRNA levels of NFE2L2, GSTA, and GCLC in the hepatocytes of APAP-treated mice. These results suggest that CRBO not only exhibited antioxidant properties in the activity levels, but this was also induced in gene expression levels. Therefore, CRBO is a promising target as an antioxidative agent.

RBO contains not only an abundance of polyunsaturated fatty acids but also a great source of phytochemicals including *γ*-oryzanols, tocotrienols, tocopherols, and phytosterols providing balancing oxidation-reduction properties with health benefits [47]. Despite having similar fatty acid profiles, WRBO contained a higher amount of *γ*-oryzanols, while levels of tocopherols, tocotrienols, phytosterols, carotenoids, and chlorophylls were higher in CRBO. According to the equal concentration of *γ*-oryzanols in the RBOs in this study, CRBO showed effective antioxidant properties. This might be due to higher levels of vitamin E, phytosterols, and plant pigments in CRBO compared with WRBO. Previous reports have shown that oral supplementation of 200 mg/kg BW of tocotrienol rich fraction (one gram contains 41.02, 6.58, 9.96, and 23.8 mg of *α*-tocotrienol, *β*-tocotrienol, *γ*-tocotrienol, and *δ*-tocotrienol, respectively) caused increased gene and protein expression of hepatic GST isoenzymes in mice [48]. Oral administration of *δ*-tocotrienol at 300 mg twice daily for 12 weeks showed greater efficacy than a placebo by decreasing oxidative stress markers including serum ALT, high-sensitivity C-reactive protein, and MDA in patients with nonalcoholic fatty liver disease [49]. Furthermore, oral feeding at 25 mg/kg BW of *β*-sitosterol modulated liver function enzymes, lipid peroxidation, and increased intracellular SOD and CAT activities in carbon tetrachloride-induced oxidative stress and liver toxicity in rats [50]. Carotenoids have also shown hepatoprotective effects by restoring the antioxidant enzymes levels in the livers of APAP-treated mice after they received 10 mg/kg BW of *β*-carotene [51]. Other plant pigments, such as 13.54 mg/kg BW of chlorophyll in 50 mg/kg BW of pigment–protein complex, could reduce the carbon tetrachloride-induced elevation of MDA, serum ALT, and AST activities and also restored suppressed hepatic SOD, CAT, and GPx activities in mice [52]. From the administration of *γ*-oryzanols at 100 mg/kg BW in each of the RBO treatment groups, CRBO-treated mice obtained higher amounts of total tocotrienol, *δ*-tocotrienol, *β*-sitosterol, carotenoids, and chlorophyll (1,702, 95.58, 9,197, 17.38, and 57.67 *μ*g/kg BW, respectively) than WRBO-treated mice (1,127, 60.16, 1,732, 9.74, and 14.33 *μ*g/kg BW, respectively). These concentrations of each compound might not be the effective doses as shown in the above reports but could synergize activity of CRBO on the amelioration of APAP toxicity. Our work suggests that CRBO could be a promising product as it is a proven antioxidant.

## 5. Conclusion

CRBO exhibited greater antioxidant potential than WRBO. The administration of CRBO attenuated hepatotoxicity induced by APAP overdose in mice through the activation of antioxidant systems and the interruption of APAP metabolism by restoring GSH and also increasing GST activity in NAPQI detoxification in the liver. The antioxidant phytonutrients and phytochemicals in CRBO included tocotrienols, phytosterols, carotenoids, and chlorophyll.

## Figures and Tables

**Table 1:** Primer sequences of qRT-PCR.

Genes	Forward primer	Reverse primer
NFE2L2	CGAGATATACGCAGGAGAGGTAAGA	GCTCGACAATGTTCTCCAGCTT
SOD1	GTGATTGGGATTGCGCAGTA	TGGTTTGAGGGTAGCAGATGAGT
CAT	GCAGATACCTGTGAACTGTC	GTAGAATGTCCGCACCTGAG
GCLC	GCACGGCATCCTCCAGTTCCT	TCGGATGGTTGGGGTTTGTCC
GSTA	CGTCCACCTGCTGGAACTTC	GCCTTCAGCAGCGGGAAAGG
GPX1	CTCACCCGCTCTTTACCTTC	CACACCGGAGACCAAATGATG
GSR	GCTATGCAACATTCGCAGATG	AGCGGTAAACTTTTTCCCATTG
*β*-Actin	GTATGACTCCACTCACGGCAAA	GGTCTCGCTCCTGGAAGATG

**Table 2:** Vitamin E, *γ*-oryzanol, phytosterols, carotenoids, and chlorophylls in RBO.

Compounds (*μ*g/g rice bran oil)	WRBO	CRBO
Vitamin E		
*α*-Tocopherol	260.2 ± 2.7	196.9 ± 2.2^∗^
Ȁ*β*-Tocopherol	28.4 ± 0.1	42.7 ± 0.2^∗^
*γ*-Tocopherol	77.7 ± 1.9	251.7 ± 1.7^∗^
*δ*-Tocopherol	Nondetectable	14.4 ± 0.3
*α*-Tocotrienol	119.6 ± 1.7	110.1 ± 1.0^∗^
*γ*-Tocotrienol	590 ± 3.9	797.5 ± 7.8^∗^
*δ*-Tocotrienol	40.0 ± 0.1	54.0 ± 0.2^∗^
Total	1,151.4 ± 10.0	1,467.2 ± 12.6^∗^
*γ*-Oryzanols		
Cycloartanyl ferulate	12,094 ± 40	8,312 ± 6^∗^
24-Methylene Cycloartanyl ferulate	12,043 ± 24	12,372 ± 11^∗^
Campesteryl ferulate	18,726 ± 97	17,709 ± 2^∗^
*β*-Sitosteryl ferulate	23,636 ± 318	18,074 ± 64^∗^
Total	66,498 ± 398	56,467 ± 57^∗^
Phytosterols		
Stigmasterol+campesterol	458 ± 2	812 ± 1^∗^
*β*-Sitosterol	1,152 ± 6	5,196 ± 9^∗^
Total	1,610 ± 8	6,008 ± 8^∗^
Carotenoids	6.48 ± 0.99	9.82 ± 0.50^∗^
Chlorophylls	9.53 ± 1.67	32.58 ± 1.24^∗^

Values are shown as mean ± SEM of three independent experiments. ^∗^Significantly different from WRBO group (*p* < 0.05).

**Table 3:** The alteration of some liver injury and oxidative stress markers in APAP-induced mice after administration of various RBOs.

Treatment	Serum ALT (U L^–1^)	Hepatic MDA (nmol mg^−1^ protein)	Hepatic total GSH (nmol mg^−1^ protein)	GSSG/GSH ratio
Vehicle	28 ± 9	0.326 ± 0.114	42.09 ± 3.75	0.31 ± 0.01
WRBO	61 ± 10^#^	0.306 ± 0.033^#^	40.25 ± 2.16^#^	0.30 ± 0.04
CRBO	52 ± 7^#^	0.291 ± 0.025^#^	42.43 ± 1.07^#^	0.30 ± 0.05
APAP	16,625 ± 5,419^∗^	0.556 ± 0.182^∗^	14.66 ± 5.17^∗^	0.27 ± 0.01
APAP+WRBO	14,082 ± 4,907^∗^	0.520 ± 0.063^∗^	11.64 ± 1.95^∗,$^	0.31 ± 0.02
APAP+CRBO	1,743 ± 617^#^	0.187 ± 0.064^∗,#^	29.26 ± 5.00^#,$^	0.29 ± 0.03
APAP+NAC	6,764 ± 1,634^#^	0.217 ± 0.096^#^	69.78 ± 8.24^∗,#^	0.28 ± 0.01

Values are represented as mean ± SEM (*n* = 6). ^∗^Significantly different from a vehicle group (*p* < 0.05). ^#^Significantly different from APAP group (*p* < 0.05). ^$^Significantly different from APAP+NAC group (*p* < 0.05). ALT: alanine aminotransferase; MDA: malondialdehyde; APAP: acetaminophen; WRBO: white rice bran oil; CRBO: coloured rice bran oil; NAC: *N*-acetylcysteine.

## Data Availability

No data were used to support this study.
